# LncRNA model predicts liver cancer drug resistance and validate *in vitro* experiments

**DOI:** 10.3389/fcell.2023.1174183

**Published:** 2023-04-03

**Authors:** Qiushi Yin, Xiaolong Huang, Qiuxi Yang, Shibu Lin, Qifeng Song, Weiqiang Fan, Wang Li, Zhongyi Li, Lianghui Gao

**Affiliations:** ^1^ Department of Hepatobiliary Surgery, The First Affiliated Hospital of Hainan Medical University, Haikou, Hainan Province, China; ^2^ Department of General, Visceral, and Transplant Surgery, Ludwig-Maximilians-University Munich, Munich, Germany

**Keywords:** CAHM, chemotherapy resistance, hepatocellular carcinoma, lncRNA, TCGA database

## Abstract

**Introduction:** Hepatocellular carcinoma (HCC) patients may benefit from chemotherapy, but drug resistance is an important obstacle to favorable prognoses. Overcoming drug resistance is an urgent problem to be solved.

**Methods:** Differential expression analysis was used to identify long non-coding RNAs (LncRNAs) that differed in chemotherapy-sensitive and chemotherapy-resistant patients. Machine learning algorithms including random forest (RF), lasso regression (LR), and support vector machines (SVMs) were used to identify important chemotherapy-related LncRNAs. A back propagation (BP) network was then used to validate the predictive capacity of important LncRNAs. The molecular functions of hub LncRNAs were investigated *via* qRT-PCR and cell proliferation assay. Molecular-docking technique was used to explore candidate drug of targets of hub LncRNA in the model.

**Results:** A total of 125 differentially expressed LncRNAs between sensitive and resistant patients. Seventeen important LncRNAs were identified *via* RF, and seven factors were identified *via* LR. With respect to SVM, the top 15 LncRNAs of AvgRank were selected. Five merge chemotherapy-related LncRNAs were used to predict chemotherapy resistance with high accuracy. CAHM was a hub LncRNA of model and expression high in sorafenib resistance cell lines. In addition, the results of CCK8 showed that the sensitivity of HepG2-sorafenib cells to sorafenib was significantly lower than that of HepG2; and the sensitivity of HepG2-sorafenib cells transfected with sh-CAHM was significantly higher than that of Sorafenib. In the non-transfection group, the results of clone formation experiments showed that the number of clones formed by HepG2-sorafenib cells treated with sorafenib was significantly more than that of HepG2; after HepG2-sorafenib cells were transfected with sh-CAHM, the number of clones formed by Sorafenib treatment was significantly higher than that of HepG2 cells. The number was significantly less than that of HepG2-s + sh-NC group. Molecular Docking results indicate that Moschus was candidate drug for target protein of CAHM.

**Conclusion:** Five chemotherapy-related LncRNAs could predict drug resistance in HCC with high accuracy, and the hub LncRNA CAHM has potential as a new biomarker for HCC chemotherapy resistance.

## 1 Introduction

Hepatocellular carcinoma (HCC) is one of the most common types of solid tumors in the world. The latest cancer statistics released in 2020 indicate that HCC is the sixth most common cancer globally, and the third most deadly (Sung et al., 2021). Annually there are reportedly 840,000 new cases of HCC and 780,000 deaths attributable to it worldwide (Ferlay et al., 2019). Currently, surgical resection, radiotherapy, chemotherapy, and immunosuppressive therapy are the main treatment methods used for HCC (Xu et al., 2018; Heller et al., 2021). Most of HCC patients require chemotherapy, but drug resistance (DR) is an important barrier that affects its efficacy in patients with malignant tumors. DR worsens prognoses, and the risk of tumor recurrence cannot be ignored, which renders the follow-up clinical treatment of patients with tumor very challenging (Shi et al., 2022). Hence, it is of the utmost importance to identify novel DR-related biomarkers for early tumor resistance detection, so as to avoid tumor resistance during drug treatment to the greatest extent possible, or to develop new machine learning models for predicting DR-related genes.

Previous studies have confirmed that DR occurs during the treatment of liver cancer, but the specific mechanisms involved are unclear. mRNA is reportedly involved in the formation of DR in liver cancer. For example, Chen et al.’s team found that the expression of CD24 was increased in drug-resistant liver cancer cells and tumor cells, and the level of CD24 was actively related to DR in liver cancer cells. They used molecular biology techniques to knock down CD24 expression in drug-resistant cells, and the cells were sensitive to sorafenib treatment, indicating that CD24 had a unique role in the phenomenon of DR in liver cancer cells (Lu et al., 2018). Other studies have shown that CD24, which is a cancer stem cell biomarker, is primarily associated with tumor resistance (Yun et al., 2016; Nakamura et al., 2017; Ishiwata et al., 2018). Metallothionein (MT) -1G is a newly discovered potential target in sorafenib-resistant HCC patients. By affecting the expression of Metallothionein (MT) -1G, sorafenib affects the sensitivity of drug treatment in patients with liver cancer. And the author made a further exploration through the mouse model, and the results were consistent with the cell level. Metallothionein (MT) -1G has a unique role in the DR phenomenon of liver cancer cells (Sun et al., 2016). In recent years, ABCC5 has been reported to be related to sorafenib resistance. At the same time, it involves postoperative chemotherapy, resulting in poor prognosis. Knockdown of ABCC5 expression in liver cancer cells significantly increased the drug sensitivity of sorafenib and reduced IC50 (Huang et al., 2021). The above studies confirm that mRNA is related to DR in HCC cells. It has also been reported that miRNAs are involved in DR in liver cancer cells, for example, Chen et al. found that miRNA-124–3p.1 levels were suppressed in sorafenib-resistant HCC cell lines, and this was associated with early recurrence in patients undergoing surgery. Similarly, they identified anti-HCC functions associated with FOXO3a and its dephosphorylation and acetylation by miR-124–3p.1, which enhanced sorafenib-induced cell death. miR-124–3p.1 can greatly improve the therapeutic effects of sorafenib *via* a dual role involving FOXO3a. Thus, the miR-124–3p.1-FOXO3a axis is considered a target for DR therapy in HCC cells (Dong et al., 2022). The above-described studies have confirmed that miRNA is associated with DR in HCC cells. It has also been reported that cirRNA plays an important role in drug resistance of liver cancer. Zhao et al. (Li et al., 2020) reported that circ_0003998 plays a role in doxorubicin (DOX) resistance in HCC, and circ_0003998 is elevated in DOX-resistant tissues and HCC cells. Knockdown of circ_0003998 using molecular biology techniques can inhibit the viability, migration, invasion, and EMT of drug-resistant cells *in vitro* and enhance the cytotoxicity of DOX *in vivo*, promoting DOX sensitivity in HCC. The above studies confirmed that CirRNA is associated with DR of liver cancer cells.

Compared with the aforementioned molecules and mechanisms, long non-coding RNAs (LncRNAs) have been comparatively less studied with respect to mechanisms of DR in liver cancer patients. In this study, a DR-related learning model was constructed to predict DR-related lncrna in patients with liver cancer. Firstly, based on TCGA-HCC data, 374 HCC patients and 50 healthy subjects were included. Differentially expressed lncrnas were obtained, and then core lncrnas were screened based on three machine learning algorithms: Random forest (RF), lasso regression (LR), and support vector machines (SVM). Finally, the predictive ability of Lncrna related to chemotherapy was obtained by BP network. Meanwhile, the prognostic value of DSS, PFI and DFS of lncrna related to chemotherapy was analyzed. The accuracy was verified by qPCR *in vitro*. The novelty of this study is that no one has used LR, SVM and RF methods to jointly analyze lncrna data in TCGA-HCC. Similarly, no one has used BP network to detect the predictive ability of chemotherapy-related lncrna. We are looking forward to identifying novel LncRNAs for the early diagnosis and treatment of DR in liver cancer, further reducing the occurrence of DR events, improving the effective drug treatment rate of liver cancer patients, and prolonging their survival.

## 2 Material and methods

### 2.1 Data acquisition and inclusion criteria

Data sourced from liver hepatocellular carcinoma (LIHC) in the public The Cancer Genome Atlas Program (TCGA) database included 374 tumor tissue gene expression profiles and 50 normal tissue gene expression profiles. Clinic information derived from each patient was downloaded from the same website. A total of 27 patients with completely therapy response information. In accordance with the research purpose, patients with clinically progressive disease or stable disease were defined as chemotherapy-resistant, and those with partial or complete responses were defined as chemotherapy-sensitive (22 cases vs. 5 cases, respectively). To qualify for enrolment in the study patients were required to have complete information pertaining to their chemotherapy procedures.

### 2.2 Analysis of differential LncRNA expression

The limma package was used to identify differential LncRNA expression in chemotherapy-resistant and chemotherapy-sensitive groups. The absolute log fold change (FC) values > 1 and *p* < 0.05 were set as the standard.

### 2.3 Machine learning to identify chemotherapy-related LncRNAs

Random forest (RF), lasso regression (LR), and support vector machines (SVM) are used to select chemotherapy related LncRNAs. We use parametric optimization, an automatic method to find best parameters of random forest model. Then set cut off value of importance to select best LncRNAs. lasso regression set criteria as log lambda equal to the minimum value, and for SVM, the AvgRank of each DE LncRNAs was calculated and according to the value, Top 15 variables will be selected, which will also be defined as important factors. After merge results of three machine learning algorithms, chemotherapy related LncRNAs will be screened.

### 2.4 Predictive capacity of chemotherapy-related LncRNAs

A back propagation (BP) network was used to test the predictive capacity of chemotherapy-related LncRNAs. First, LncRNA expression was assigned a value of “0” or “1” based on the median value of each chemotherapy-related LncRNA. Chemotherapy-resistant and chemotherapy-sensitive groups were then generated. This matrix was entered into an R program to construct a BP network, and the area under the curve (AUC) was calculated to evaluate the predictive capacity of chemotherapy-related LncRNAs in patients who were chemotherapy-resistant and chemotherapy-sensitive.

### 2.5 Identifying hub LncRNA and co-expression of different mRNAs

Each chemotherapy-related LncRNA was assigned an importance value based on RF results, then ordered to identify hub LncRNAs. Differential expression analysis of mRNAs in resistant and sensitive groups was then performed, and R code was used to conduct batch co-expression analysis between hub LncRNAs and DE mRNAs. The absolute value of correction was set at 0.1.

### 2.6 Function enrichment of hub LncRNA target mRNAs

To investigate candidate functions of hub LncRNAs, co-expression of mRNAs was enriched *via* Gene oncology and Reactome Pathway analyses. Enrichment results with *p* values < 0.05, were selected.

### 2.7 Hub LncRNA survival analysis using a public dataset

Prognostic value of hub LncRNAwas also considered important for patients, and survival analysis of hub LncRNAs was performed with respect to progression-free interval (PFI), disease-specific survival (DSS), and overall survival (OS), and Disease -free interval (DFI).

### 2.8 Cell transfection

Genema Biotechnology Co., Ltd. (Shanghai, China) provided sh-CAHM and its negative control sh-NC. Lipofectamine 2000 reagent (Invitrogen, Carlsbad, CA, United States) was used for transfection, following the manufacturer’s instructions. The HepG2-sorafenib-resistant cells were transfected with either sh-NC or sh-CAHM, and the resulting groups were named HepG2-s + sh-NC and HepG2-s + sh-CAHM, respectively.

### 2.9 qRT-PCR

The liver cancer cell line used in this study was HepG2. Cells were cultured in DMEM. Primer information of CAHM is shown in (Table 1). qPCR was used to test whether hub LncRNA expression differed in liver cancer cells and chemotherapy-resistant cell lines. Total RNA was extracted from each group of cells and tissues using TRIZOL (Invitrogen, Carlsbad, CA, United States), sample concentration and purity were verified after extraction, and reverse transcription was performed using a reverse transcription kit (TaKaRa, Tokyo, Japan). Gene expression was measured using a LightCycler 480 (Roche, Indianapolis, IN, United States) fluorescence PCR instrument in accordance with the operating conditions described in the fluorescence PCR kit (SYBR Green Mix, Roche Diagnostics, Indianapolis, IN, United States). Data analysis was performed using the 2^−ΔΔCT^ method.

**TABLE 1:** primer sequence of LncRNA CAHM.

Name	Primer	Sequence	Size
Homo GAPDH	Forward	TCA​AGA​AGG​TGG​TGA​AGC​AGG	115bp
	Reverse	TCA​AAG​GTG​GAG​GAG​TGG​GT	
Homo CAHM	Forward	GCA​CCT​TCC​CGT​TAT​TCT​GA	163bp
	Reverse	GCG​CAC​AGC​CTC​TTT​ATT​TC	

### 2.10 CCK8 assay

To determine the dose-response of liver cancer cells to sorafenib, the cells were digested and seeded in a 96-well plate at a density of 5,000 cells per well. Three wells were seeded for each sample, and different concentrations of sorafenib (0 μM, 1 μM, 2 μM, 5 μM, 10 μM, and 20 μM) were added to each well. After 24 h of incubation at 37°C, 10 μL of CCK8 reagent (Tokyo, Dojindo, Japan) was added to each well. After further incubation for 2 h at 37°C, the absorbance was measured at 450 nm wavelength.

### 2.11 Clonogenic assay

The cells were collected and digested with trypsin, centrifuged at 25°C and 1,500 rpm for 5 min, and resuspended in complete medium. 500 cells were seeded in a 6-well plate containing 2 mL of preheated complete medium at 37°C. The sorafenib-treated group was added with 10 μM sorafenib, and the control group was added with an equal amount of DMSO. The cells were cultured under 37°C and 5% CO_2_ conditions. When visible cell clones were observed in the 6-well plate, the culture was terminated. The cells were washed twice with PBS, fixed with 1.5 mL of methanol for 15 min, and then stained with 1 mL of Giemsa staining solution for 20 min. The number of clones was counted after washing the plate with running water and drying it on a clean absorbent paper.

### 2.12 Select hub targets of LncRNA CAHM and predict related candidate small molecular drugs

To identify the hub targets of LncRNA CAHM, we use string database (https://string-db.org/) to conduct protein-protein interaction (PPI) network analysis of the above positive related genes. During analysis, disconnect proteins will be hidden. In addition, The MCODE plug was used to identify the hub protein cluster. After performing this operation we use the Symmap database (http://www.symmap.org/) to predict hub proteins related to herbs, then merge all of these predictive drugs according to each target to select the intersecting herbs.

As herbs including different elements, the first step, we use TCMSP database to identify key small molecular and then download small molecular structure and protein structure from PubChem (https://pubchem.ncbi.nlm.nih.gov/) and PDB (https://www.rcsb.org/), respectively. Auto dock tools will be performed to dock hub targets and small molecular drugs.

## 3 Results

### 3.1 Workflow and DE LncRNAs in sensitive and resistant groups

**FIGURE 1:**
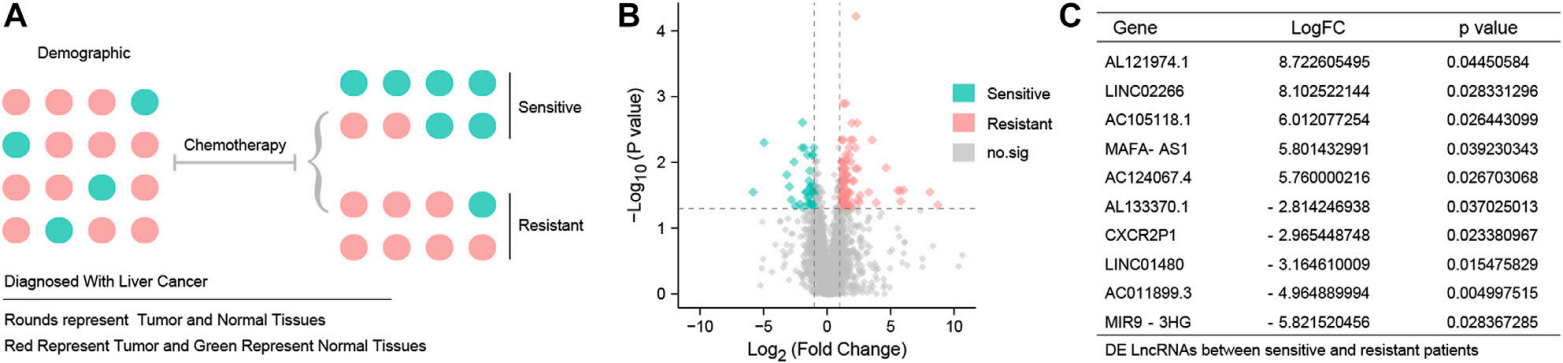
Different expression LncRNAs between resistant and sensitive patients **(A)** TCGA-HCC patients were grouped according to chemotherapy sensitivity. Red represents tumor patients and green represents normal subjects. **(B)** Volcano map of 125 DE LncRNAs. Red point represents 88 up-DE LncRNAs, green point represents 37 down-DE LncRNAs, and gray point represents DE LncRNAs with no statistical significance. The threshold is set to log fold change (FC) values > 1 and *p* < 0.05. **(C)**10 DE LncRNAs with the most significant fold change were AL121974.1, LINC02266, AC105118.1, MAFA-AS1, AC124067.4, AL133370.1, CXCR2P1, LINC01480, AC011899.3, and MIR9-3HG.

Figure 1A shows a detailed representation of the research procedure used to identify patients who would benefit from chemotherapy and those who would be chemotherapy-resistant. Differential expression analysis identified a total of 125 DE LncRNAs in sensitive and resistant patients, including 88 upregulated LncRNAs and 37 downregulated LncRNAs. A volcano map is shown in Figure 1B. Sort gene expression by log fold change (FC) value**,** and the top 10 DE LncRNAs are shown in Figure 1C.

### 3.2 Selection of five chemotherapy-related LncRNAs

**FIGURE 2:**
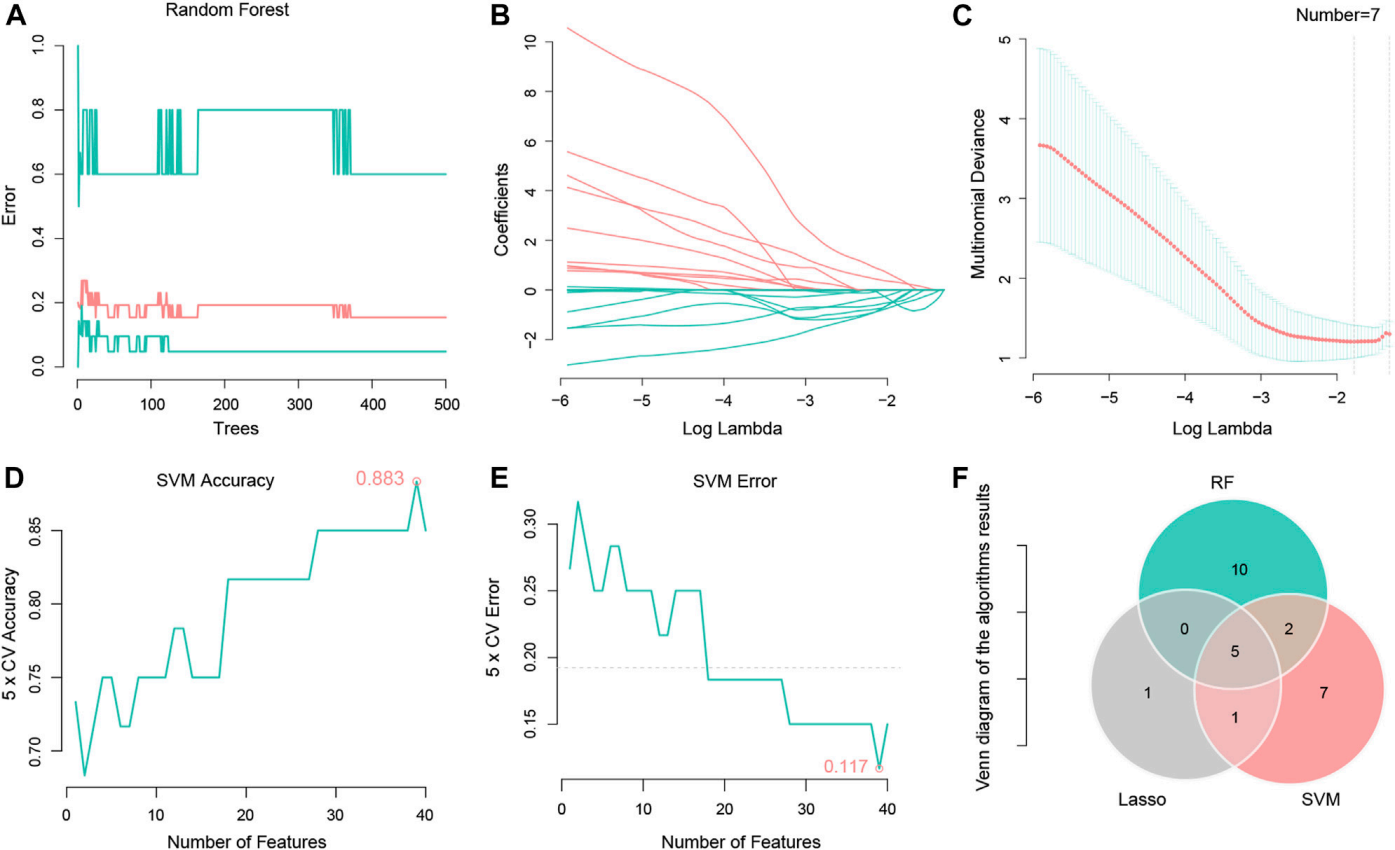
Using Random forest, Lasso regression and SVM algorithms to identify five chemotherapy related LncRNAs. **(A)** Random forest tree model, the number of random forest trees is set to 500 (horizontal coordinate). The vertical coordinate represents the cross validation error. The bottom green line represents the error of the control group, the red line represents the error of all the samples, and the top green line represents the error of the experimental group. **(B)** LASSO regression of the 7 genes. **(C)** The abscissa represents logλ, and the ordinate represents the cross validation error. The point with the least validation error corresponds to 7 genes. **(D)** A graph representing the accuracy of the SVM. The figure shows an accuracy of 0.883. **(E)** Graph representing SVM cross validation error, using 5X cross validation, error shown in the figure is 0.117. **(F)** Venny diagram shows 5 intersection genes of the 3 algorithms, respectively LINC01857, AC064836.3, CAHM, AL691432.1, and GBP1P1.

The RF set mtry value was 2, the number of trees was 500, the model obtained the best AUC, then the model output all importance values of the variables. When importance was set to > 0.1, seventeen important LncRNAs were identified (Figure 2A). An LR model was also used to select important factors. When log lambda obtained the minimum value, a total of seven factors were output (Figures 2B, C). Another machine learning algorithm, SVM, was used to calculate AvgRank and select the top 40 genes to test the model. The accuracy of the model was 0.883, and the error of the model was 0.117 (Figures 2D, E). This result demonstrated that the model was robust. The top 15 LncRNAs were then selected based on model results, and merged with results from the other two machine learning algorithms, Lastly, five chemotherapy-related LncRNAs were obtained (Figure 2F). Five chemotherapy - related lncrna were LINC01857, AC064836.3, CAHM, AL691432.1, and GBP1P1.

### 3.3 Five chemotherapy-related LncRNAs could predict the effects of chemotherapy

**FIGURE 3:**
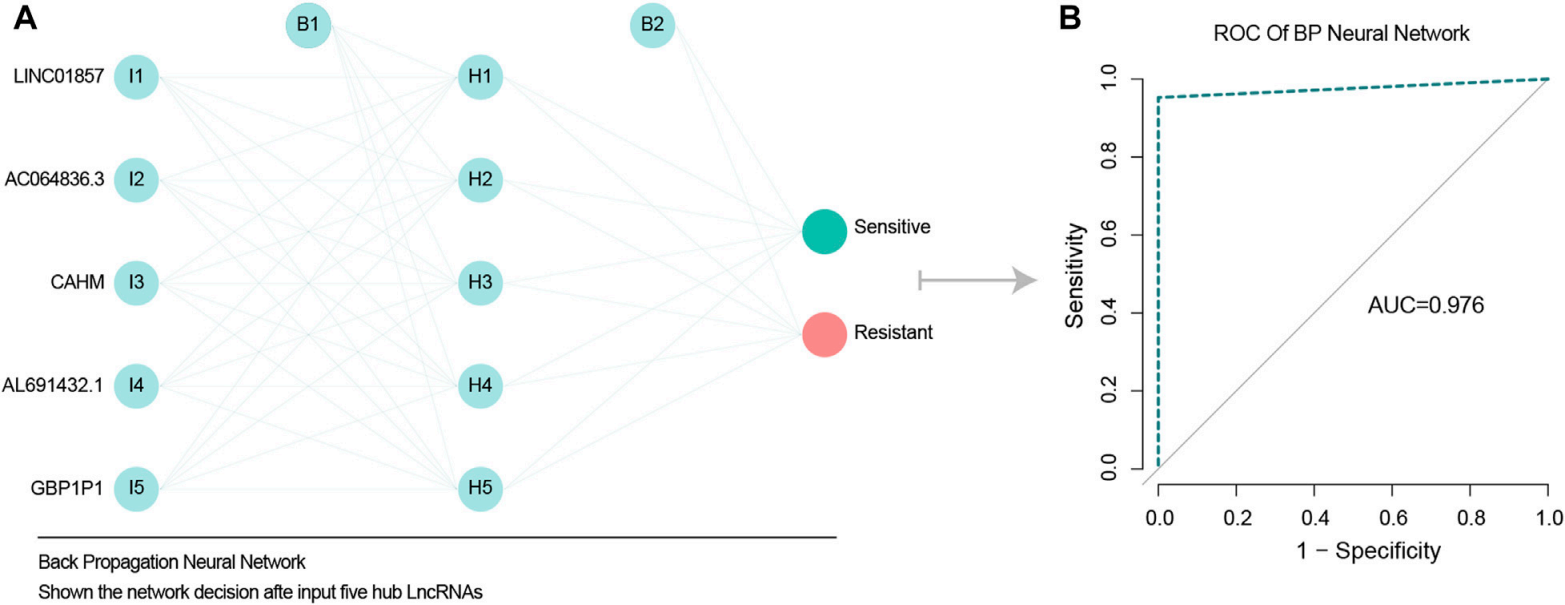
BP network validate predict ability of five chemotherapy related LncRNAs. **(A)** BP neural network diagram, shown from left to right respectively are the input layer, hidden layer 5 nodes, output layer. **(B)** ROC model verified the accuracy of BP network, and AUC value was 0.976, indicating high accuracy of BP network.

A BP network was used to assess whether the five chemotherapy-related LncRNAs identified could predict the effects of chemotherapy. The results indicated that they could do so with high accuracy, and an AUC of 0.976 (Figures 3A, B).

### 3.4 CAHM as a hub LncRNA involved in important pathways

**FIGURE 4:**
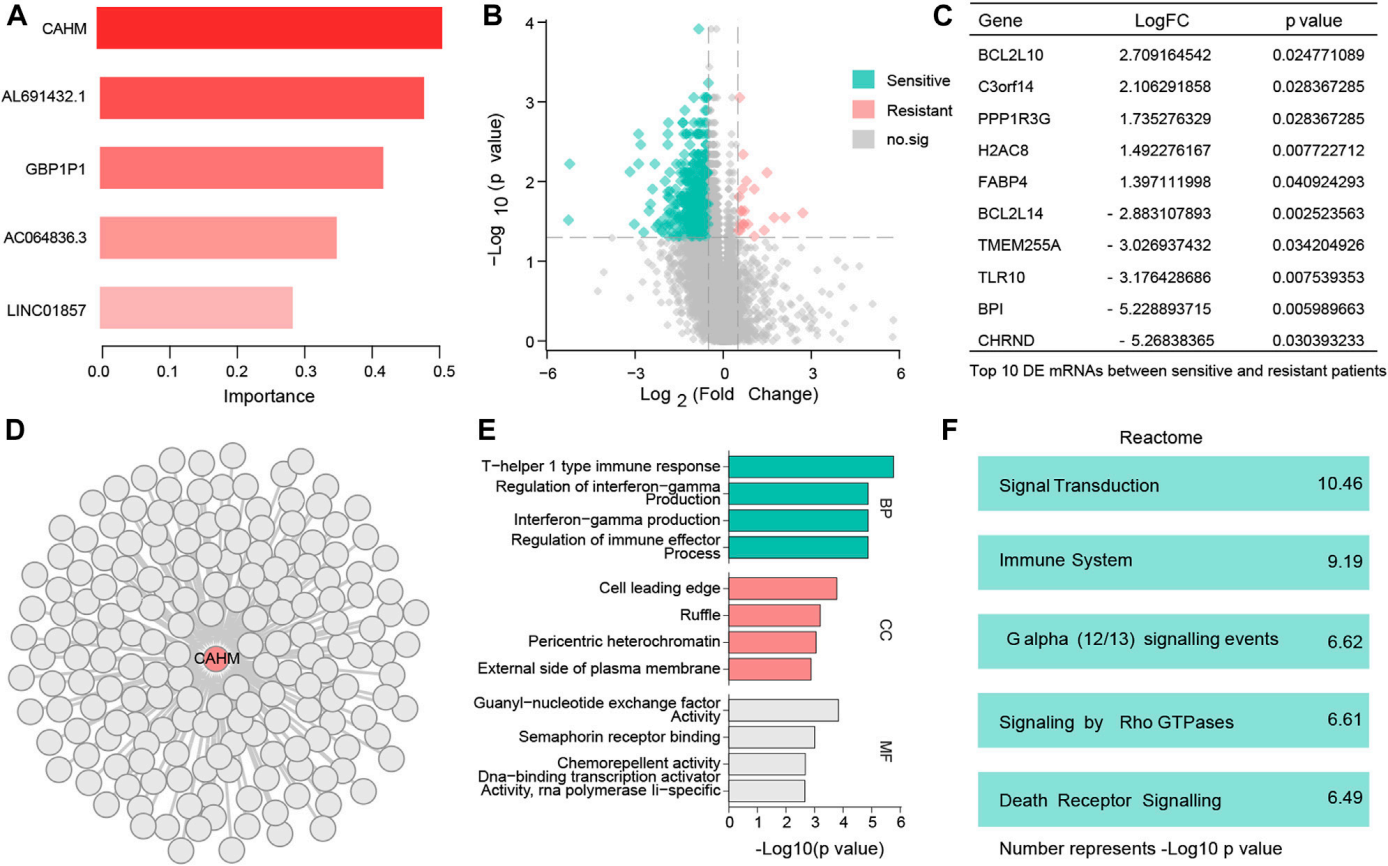
Identify hub LncRNA CAHM and explore molecular function of this LncRNA **(A)** Bar chart shows 5 resistant genes. **(B)** 652 DE mRNAs were identified. **(C)** Top 10 DE mRNAs with the largest change multiples. **(D)** 181 DE mRNAs were positively correlated with CAHM. **(E)** Gene Ontology (BP, CC, and, MF) analysis of CAHM. **(F)** Reactome analysis of CAHM.

In RF analysis, of the five chemotherapy-related LncRNAs CAHM had the largest importance value, so it was defined as a hub chemotherapy-related LncRNA (Figure 4A). Candidate co-expressed mRNAs among the DE mRNAs were investigated in the resistant and sensitive groups, and 652 DE mRNAs were identified (Figure 4B). The top 10 DE mRNAs are shown in Figure 4C. A total of 181 DE mRNAs were positively correlated with CAHM (correlation coefficient > 0.1) (Figure 4D). Results obtained by inputting these genes into the online KOBAS tool to investigate molecular functions of CAHM indicated that they were enriched in cell leading edge, and involved in signal transduction pathways (Figures 4E, F).

### 3.5 High expression of CAHM is associate with poor outcome

**FIGURE 5:**
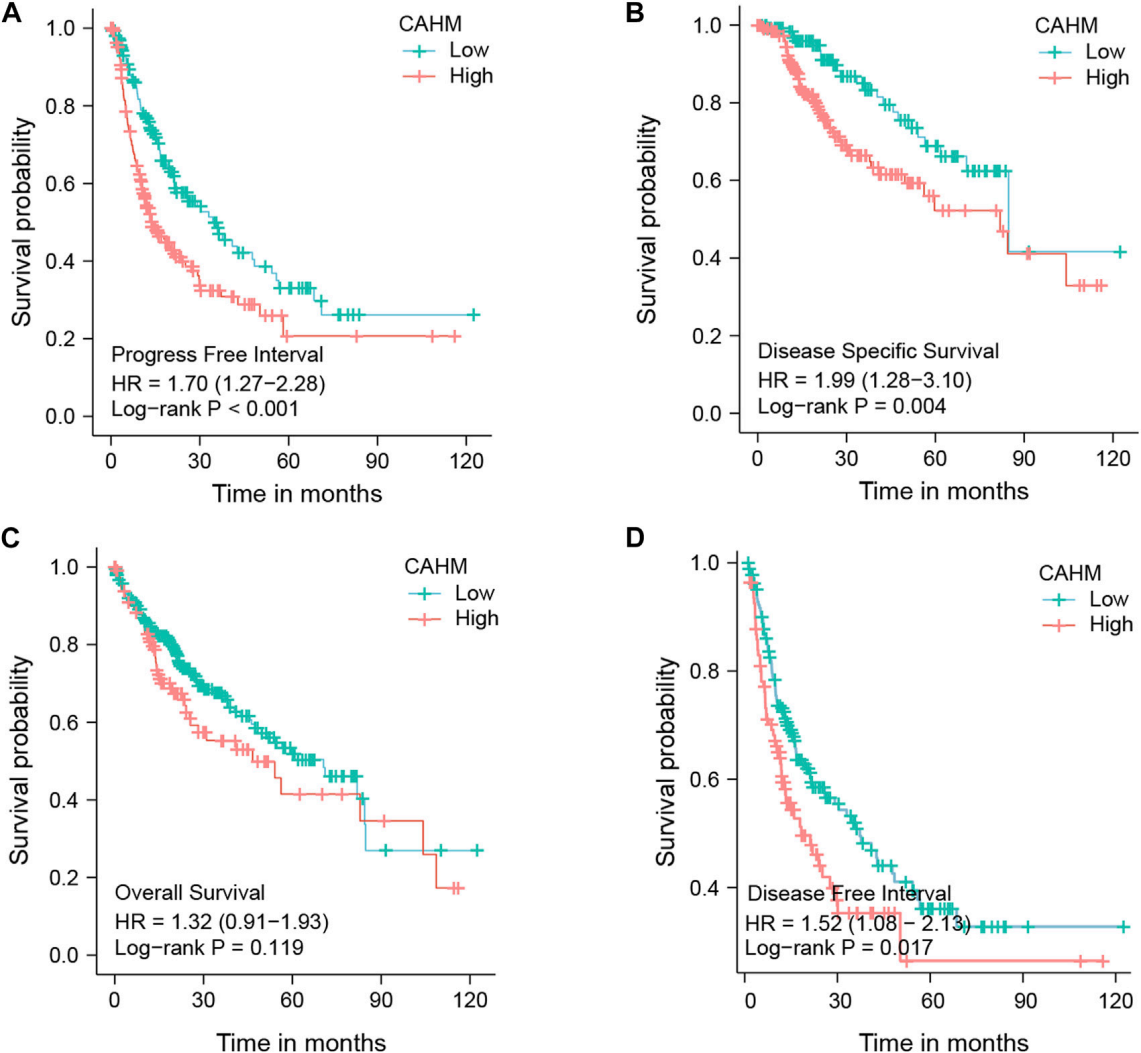
Survival analysis of hub LncRNA CAHM. **(A)** Survival analysis of PFI. **(B)** Survivla analysis of DSS. **(C)** Survival analysis of OS. **(D)** Survival analysis of DFI.

Survival analysis results from public database show that, compared with low expression of CAHM, high expression CAHM patients with a poor outcome, while in PFS,DSS, and OS, and DFI group (*p* < 0.001, *p* = 0.004, *p* = 0.119, and *p* = 0.017; respectively) (Figures 5A–D). It is worth noting that, although, in OS group the *p*-value was no significant, the trend is also support high expression CAHM patients with a poor outcome.

### 3.6 qRT-PCR and cell proliferation

**FIGURE 6:**
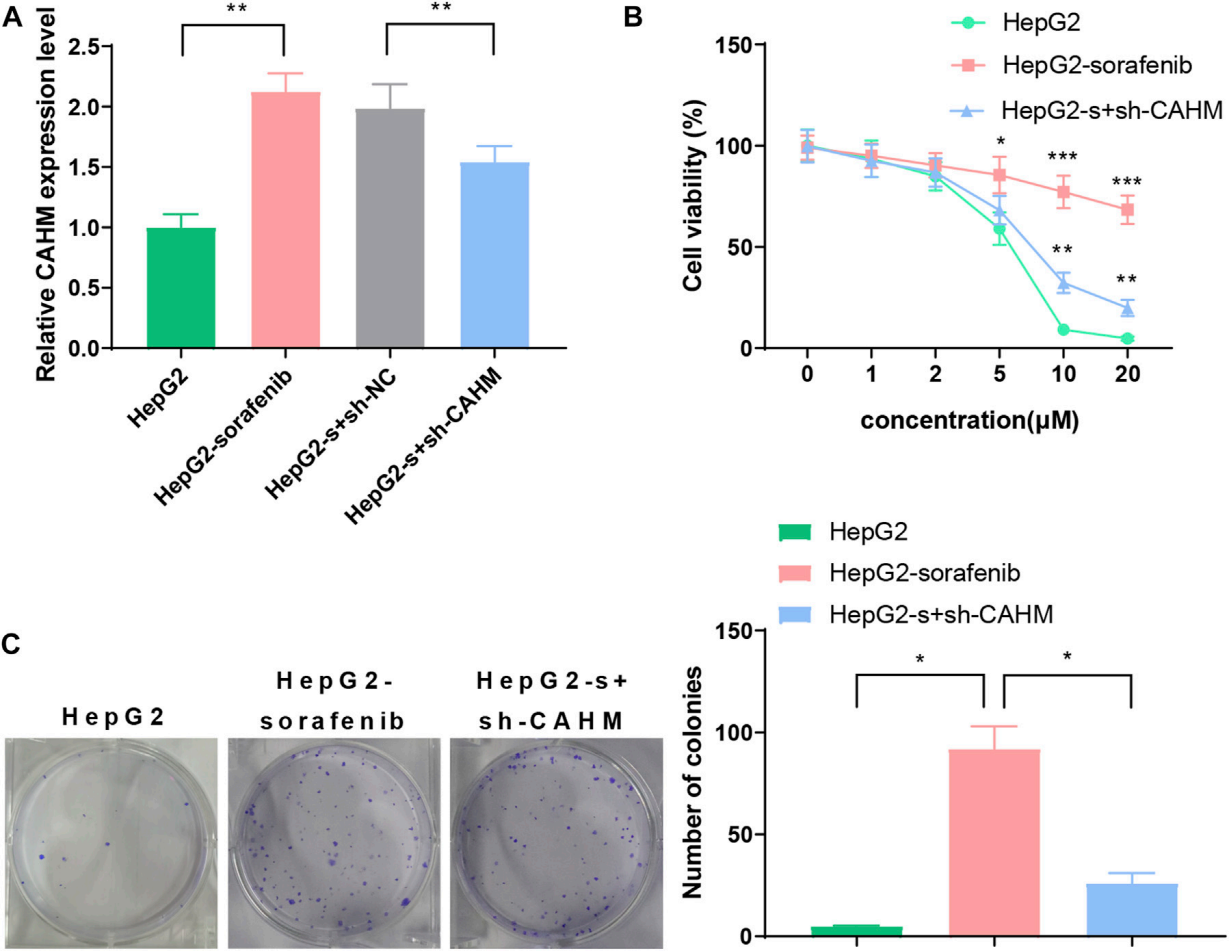
CAHM expression and cell proliferation **(A)** qRT-PCR validate CAHM expression in HepG2-sorafenib group was higher than that in the HepG2 group. **(B)** CCK8 assay show that the sensitivity of HepG2-sorafenib cells to sorafenib was significantly lower than that of HepG2 cells **(C)** Clonogenic assay demonstrate that the number of clones formed by sorafenib-treated HepG2-sorafenib cells was significantly higher than that of HepG2 cells.

The qRT-PCR results revealed that the expression level of CAHM in the HepG2-sorafenib group was higher than that in the HepG2 group. Transfection of sh-CAHM into HepG2-sorafenib cells downregulated the expression of CAHM, indicating that the HepG2-s + sh-CAHM group significantly downregulated CAHM expression compared to the HepG2-s + sh-NC group (Figure 6A). It is worth noting that according to above experiment, there was no statistical difference in the expression of CAHM between the HepG2-sorafenib group and the HepG2-s + sh-NC group, indicating that transfection of sh-NC in HepG2-sorafenib cells had no effect on the expression of CAHM, the HepG2-s + sh-NC group was omitted in subsequent experiments. The CCK-8 experiment results showed that the sensitivity of HepG2-sorafenib cells to sorafenib was significantly lower than that of HepG2 cells. However, after transfection of sh-CAHM into HepG2-sorafenib cells, the sensitivity to sorafenib was significantly increased compared to the non-transfected group (Figure 6B). The clonogenic assay results showed that the number of clones formed by sorafenib-treated HepG2-sorafenib cells was significantly higher than that of HepG2 cells. Nevertheless, after transfection of sh-CAHM into HepG2-sorafenib cells, the number of clones formed after sorafenib treatment decreased to less than 5% of the total number of cells seeded (Figure 6C).

### 3.7 Moschus was a candidate small molecular for HCC

**FIGURE 7:**
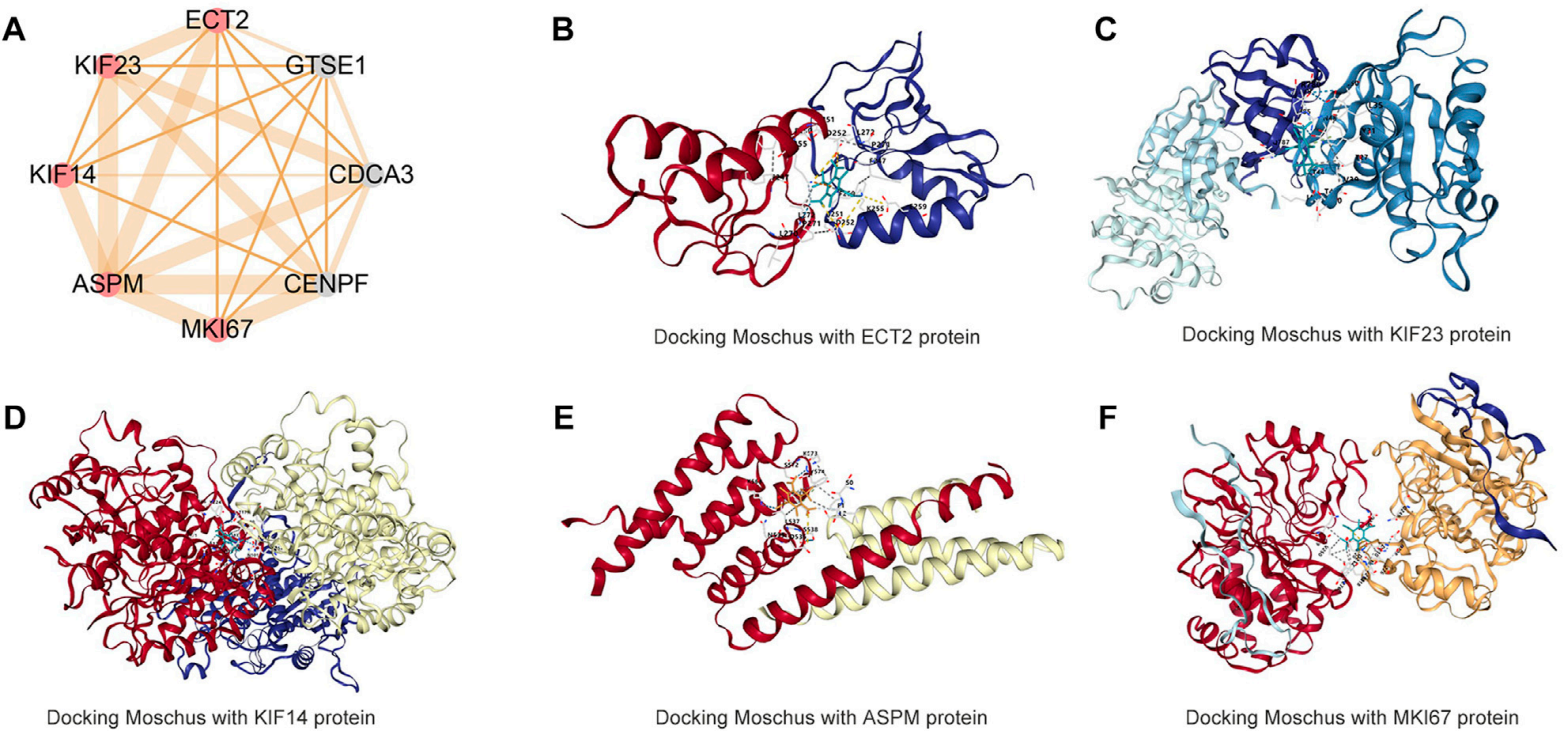
Molecular docking Moschus with hub targets of CAHM **(A)** hub targets of CAHM *via* MCODE algorithms **(B)** Docking Moschus with ECT2 protein. **(C)** Docking Moschus with KIF23 protein. **(D)** Docking Moschus with KIF14 protein. **(E)** Docking Moschus with ASPM protein. **(F)** Docking Moschus with MKI67 protein.

The PPI network shows that most proteins connect and show strong protein interactions (Supplementary Figure S1) and the MCODE results show cluster one includes eight hub genes. However, three proteins with no structure were excluded (Figure 7A). The other five proteins are included and related to small molecular drugs predicted successfully (Supplementary Tables S1–S5). The merge results show that only two drugs are common, they are Cervi Cornu Pantotrichum and Moschus. Cervi Cornu Pantotrichum with no report for liver cancer, which means a lack of clinical evidence, so, we select Moschus as a candidate drug, the docking result shows that five hub proteins bind tightly to the drug (Figures 7B–F).

## 4 Discussion

The incidence of DR in cancer patients undergoing clinical treatment is increasing, and this has become a challenging phenomenon. Machine learning is a branch of artificial intelligence. It has unique advantages for dealing with clinical and scientific data, so it is becoming more widely used in various fields of scientific research. SVM algorithms are supervised machine learning algorithms widely used in bioinformatics (Ng and Mishra, 2007). The core idea of classification is to identify an optimal classification hyperplane between two classes of samples, so that input data can be optimally classified after being mathematically transformed into high-dimensional space. Its advantages include applicability to small samples, non-linear data, and high dimensional data, and it can also facilitate informative statistical analyses (Maglogiannis et al., 2009). RF can enhance the performance of risk prediction by using large data repositories to identify new risk predictors and more complex interactions between them. Its advantage is that it can process very high-dimensional data. Feature subsets are randomly selected to obtain feature importance. The training speed is fast and easy to parallelize (Rigatti, 2014; AlaaID et al., 2019). LASSO, also known as the minimum absolute value convergence and selection operator algorithm, performs variable selection and complexity adjustment while fitting the generalized linear model. Therefore, whether the dependent variable is continuous, binary or multivariate discrete, it can be modeled and predicted by LASSO regression (Guler and Guler, 2021; Kang et al., 2021; Mullah et al., 2021). The objective of the current study was to use machine learning methods to identify novel biomarkers of DR events in liver cancer patients undergoing clinical treatment, to obtain a deeper understanding of the biological mechanisms involved, expand the knowledge-base with respect to of DR genes, and provide a fresh direction ultimately aimed at curing patients with clinical DR.

Signal transduction pathways are associated with a variety of biological processes. Similarly, signal transduction pathways also include a variety of. For example,: apoptosis related signaling pathway, immune related signaling pathway, G alpha 12/13 signaling pathway, etc. Among them, the apoptosis pathway is related to tumor cell death, and chemotherapy drugs promote tumor cell apoptosis and improve the prognosis of patients. We found that CAHM may be involved in apoptosis-related pathways. The G 12/13 pathway mediates calcium-independent shape changes and plays an enhanced role in Akt phosphorylation and dense granule secretion. It also affects platelet function (Bhavaraju et al., 2011). Akt plays a role in many tumor biological processes, and the G 12/13 pathway is related to AKT, indicating that CAHM may regulate tumor-related biological processes by regulating AKT phosphorylation or dephosphorylation.

At present, reports on CAHM are relatively scarce. CAHM was previously named LOC100526820, it is located on chromosome 6, hg19 chr6:163 834 097–163 834 982, and it is currently categorized as an LncRNA. The gene is considered not to encode a protein, but can encode an LncRNA, RefSeq NR_037593, now renamed LINC00468. LncRNAs evidently play both carcinogenic and tumor suppressor roles (Bhan et al., 2017). They are involved in tumor initiation, tumor progression, and distal tumor metastasis, and they have been implicated in dysregulation of related gene expression and signaling pathways (Slack and Chinnaiyan, 2019). The LncRNA CAHM reportedly has unique roles in the biological progression of many tumors, including breast cancer, glioma, colorectal cancer, thyroid cancer, and liver cancer. For example, Xie et al. (Li et al., 2022) obtained mRNA and LncRNA data derived from breast cancer patients in a public database (Gene Expression Omnibus), then used the WGCNA analysis method to construct a relevant module for identification of different subtypes of breast cancer. They then set the absolute Pearson correlation coefficient to > 0.6, and constructed an lncRNA-mRNA network. They reported that CAHM could be used as a positive predictor of breast cancer survival probability, indicating that it can be used as a potential biomarker in the treatment and prognostic classification of different subtypes of breast cancer. The above studies have shown that CAHM plays a role in the occurrence and development of breast cancer. Similarly, Zhao et al. showed that CAHM expression was associated with glioma grade, molecular subtype, IDH mutation status, and 1q/19p coding status. Gene ontology analysis showed that CAHM was involved in cell development, nervous system development, neurogenesis, and integrin-mediated signaling pathways. CAHM overexpression inhibited glioma cell proliferation, colony formation, and invasion. In another study CAHM overexpression inhibited glioma migration and invasion *via* the SPAK/MAPK pathway. Interestingly, in that study CAHM expression was negatively correlated with OS in primary and recurrent gliomas (Xu et al., 2022). Thus, CAHM plays a role in biological processed involved in glioma.

Molloy et al. reported that CAHM methylation level was associated with colon cancer progression. CAHM methylation levels were significantly higher in tumor tissues (adenoma and colon cancer) than in normal colon tissues. With 5% methylation as the threshold, 2/26 normal tissues (8%), 17/21 adenomas (81%), and 56/79 cancers (71%) were positive. The proportion of LncRNA-CAHM methylation in CRC stage II–IV was significantly higher than that in normal colorectal specimens (*p* < 0.0002), and the median CAHM methylation level in tumor colorectal specimens was 17%–40%. The authors also detected high methylation levels of CAHM in breast cancer (Pedersen et al., 2014). The above studies indicate that CAHM methylation is associated with colon tumor progressions. CAHM methylation is also associated with breast cancer. Similarly, CAHM plays a role in thyroid cancer. Li et al. reported that in THCA patients the risk of death in those with high CAHM expression was lower than the risk of death in those with low CAHM expression (log rank *p* = 0.022, adjusted *p* = 0.011, hazard ratio 0.187, 95% confidence interval 0.051–0.685). CAHM acts as a protective factor in thyroid carcinoma. Li et al. screened five drugs (levobuprolol, NU-1025, quinprazine, anisomycin, and sulfathiazole) to target the CAHM gene in THCA patients (Xiao et al., 2021).

Of course, the study of this manuscript has certain limitations. In recent years, with the development of sequencing and chip technology, the molecular mechanisms of more and more diseases are more clearly understood. Most patients can benefit from the development of these technologies. However, there are still many deficiencies. Biopsy of the tumor, sequencing, and then guiding the treatment of the patient according to the mutation, however, there are few new advances in clinical treatment. At the same time, sequencing is expensive, increasing the burden of treatment on patients, and so on. Finally, even if the sequencing was carried out, the targeted drugs of the target gene were screened, but the patients did not benefit from the treatment because of the problem of drug sensitivity.

In conclusion, the above studies show that five chemotherapy-related LncRNAs can predict DR in liver cancer with high accuracy, and the hub LncRNA CAHM has the potential to become a new biomarker for chemotherapy-resistant liver cancer.

## Data Availability

Publicly available datasets were analyzed in this study. This data can be found here: https://portal.gdc.cancer.gov/.

## Supplementary material

SUPPLEMENTARY FIGURE S1 PPI network analysis of positive correlation genes of LncRNA CAHM.

SUPPLEMENTARY TABLE S1 Predict candidate small molecules of ECT2.

SUPPLEMENTARY TABLE S2 Predict candidate small molecules of KIF23.

SUPPLEMENTARY TABLE S3 Predict candidate small molecules of ASMP.

SUPPLEMENTARY TABLE S4 Predict candidate small molecules of KIF14.

SUPPLEMENTARY TABLE S5 Predict candidate small molecules of MKI67.

## Abbreviations

AUC, Area under the curve; BP, Back propagation; circRNA, Circular RNA; DE LncRNAs, Differentially expressed LncRNAs; DFI, Disease-free interval; DOX, Doxorubicin; DR, Drug resistance; DSS, Disease-specific survival; FC, Fold change; GEO, Gene Expression Omnibus; HCC, Hepatocellular carcinoma; LIHC, Liver Hepatocellular carcinoma; LncRNAs, Long non-coding RNAs; LR, Lasso regression; OS, Overall survival; PFI, Progression-free interval; RF, Random forest; SVM, Support vector machines; TCGA, The Cancer Genome Atlas.
