# Supplementary figures and images for: LncRNA model predicts liver cancer drug resistance and validate in vitro experiments
Source: Front Cell Dev Biol. 2023 Apr 3;11:1174183. doi: 10.3389/fcell.2023.1174183 (PMC10106610; doi:10.3389/fcell.2023.1174183)

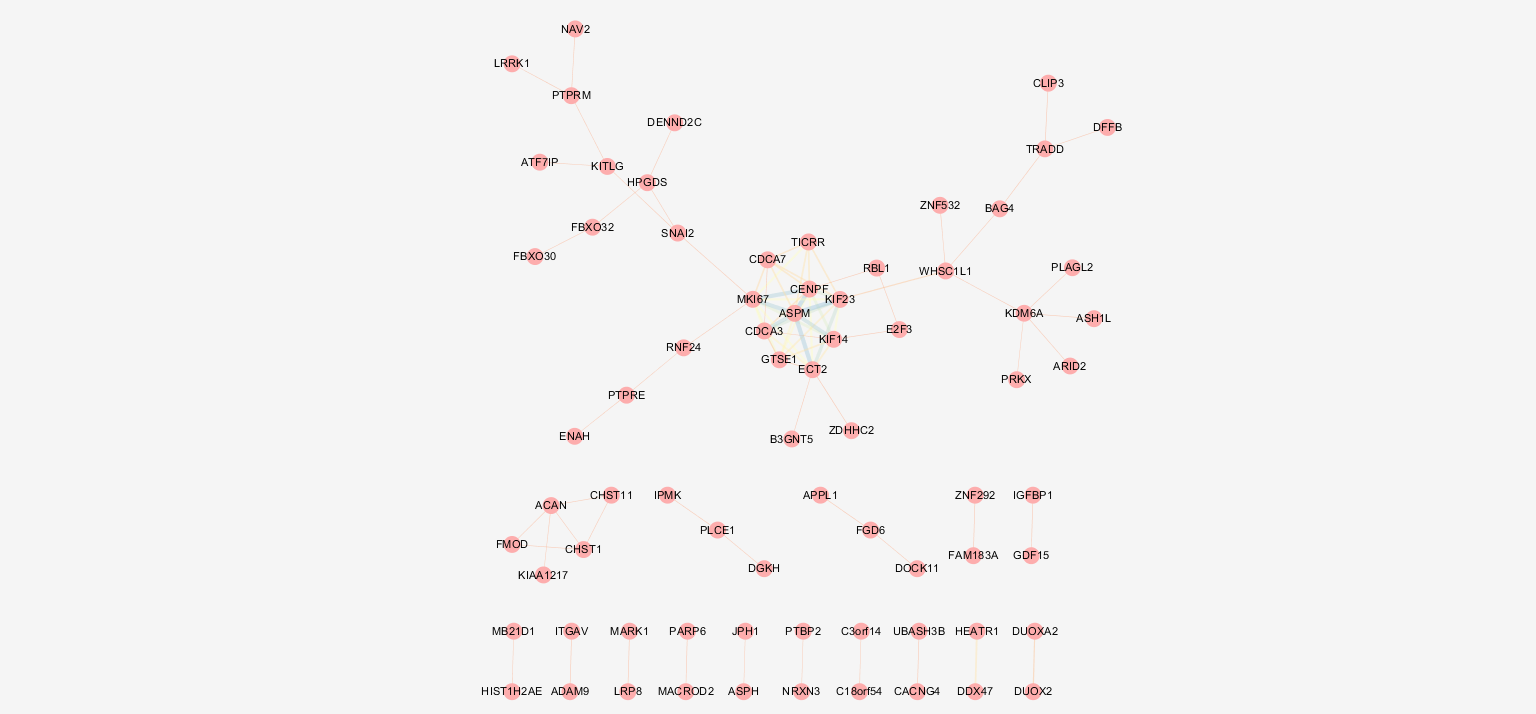

Supplement: Supplementary file 3 [file Image1.PNG]
